# Evaluation of Risk Factors for Postbooster Omicron COVID-19 Deaths in England

**DOI:** 10.1001/jamanetworkopen.2022.33446

**Published:** 2022-09-08

**Authors:** Vahé Nafilyan, Isobel L. Ward, Chris Robertson, Aziz Sheikh

**Affiliations:** 1 Health Analysis and Life Events Division, Office for National Statistics, Newport, United Kingdom; 2 Department of Mathematics and Statistics, University of Strathclyde, Glasgow, United Kingdom; 3 Public Health Scotland, Glasgow, United Kingdom; 4 Usher Institute, University of Edinburgh, Edinburgh, United Kingdom

## Abstract

This cohort study of adults in England evaluates the association of sociodemographic and clinical risk factors with death from COVID-19 among individuals who completed primary vaccination and received a messenger RNA (mRNA) booster.

## Introduction

With the emergence of the Omicron variant, it has become critical to identify risk factors associated with COVID-19 death in individuals who have completed primary vaccination and received a messenger RNA (mRNA) booster dose. Existing evidence is based on people who have received 1 or 2 doses of a COVID-19 vaccine and were infected by the Alpha or Delta variant.^1,2,3^ Understanding which groups are at increased risk of COVID-19 death after receiving a booster is crucial for the prioritization of further booster doses and access to COVID-19 therapeutics.

## Methods

We used data from the Office for National Statistics Public Health Data Asset, a population-level linked data set combining the 2011 Census of England and Wales and electronic health records covering 80% of the population of England (eMethods in the Supplement). Ethics approval was obtained from the National Statistician’s Data Ethics Advisory Committee and reporting follows the Strengthening the Reporting of Observational Studies in Epidemiology (STROBE) guideline for cohort studies. Our study population included those aged 18 to 100 years living in England who had completed both doses of their primary vaccination schedule and had received their mRNA booster 14 days or more prior to December 31, 2021.

Our outcome of interest was time to death involving COVID-19 (ie, *International Statistical Classification of Diseases and Related Health Problems, Tenth Revision* codes U071 and U072 recorded on death certificate) occurring between January 1 and March 16, 2022. Cox regression modeling (R version 3.5.1 [R Project for Statistical Computing]) was used to estimate the hazard ratio (HR) and 95% CIs of death involving COVID-19 in 2-tailed tests for a range of sociodemographic characteristics (sex, age, ethnicity, and measures of socioeconomic position) and clinical risk factors (based on the conditions identified in the QCovid risk model [eTable in the Supplement]).^1^ Data on ethnicity from the 2011 Census used the selected categories identified by ONS, which were aggregated to the UK Government Statistical Service harmonized standard. Non–COVID-19 deaths were censored. Models were adjusted for time since booster dose as of December 31, 2021, and region.

## Results

In the 19 473 570 individuals in our cohort (8 801 865 men [45.2%]; mean [SD] age, 60.8 [16] years; 17 918 962 [92.0%] White individuals), there were 4781 (0.02%) deaths involving COVID-19 and 58 020 (0.3%) deaths from other causes. The median (IQR) age of those who died of COVID-19 was 85 (78-99) years (mean, 83.3 years) (Table).

**Table.** Characteristics of the Study Population and Deaths

Variable	Individuals, No. (%)	COVID-19 deaths, No. (%)
Deaths	62 801 (0.3%)	4781 (0.02)
Mean (SD) age, y	60.8 (16)	83.3 (9.8)
Sex		
Men	8 801 865 (45.2)	2664 (55.7)
Women	10 671 705 (54.8)	2117 (44.3)
Mean (SD) time since booster dose, d	48 (24)	66.2 (21.8)
Race and ethnicity		
Bangladeshi and Pakistani	223 712 (1.1)	35 (0.7)
Black	275 350 (1.4)	37 (0.8)
Chinese	87 800 (0.5)	10 (0.2)
Indian	495 847 (2.5)	95 (2.0)
White	17 918 962 (92.0)	4566 (95.5)
Mixed	170 114 (0.9)	11 (0.2)
Other	301 785 (1.5)	27 (0.6)
IMD		
1 (most deprived)	2 671 942 (13.7)	900 (18.8)
2	3 384 141 (17.4)	898 (18.9)
3	4 103 685 (21.1)	1011 (21.1)
4	4 520 346 (23.2)	980 (20.5)
5 (least deprived)	4 793 456 (24.6)	992 (20.7)
Key worker status	12 781 562 (65.6)	3252 (68.0)
Disability (limited a lot)	1 307 789 (6.7)	1176 (24.6)
Education: degree	6 015 501 (30.9)	757 (15.8)
Asthma	1 340 726 (6.9)	288 (6.0)
Atrial fibrillation	649 076 (3.3)	771 (16.1)
Cancer of blood or bone marrow	134 668 (0.7)	266 (5.6)
Chronic kidney disease	728 667 (3.7)	874 (18.3)
COPD	681 708 (3.5)	708 (14.8)
Congenital heart problem	28 187 (0.1)	<10^a^
Coronary heart disease	685 197 (3.5)	572 (12.0)
Other rare chronic respiratory disorders (cystic fibrosis bronchiectasis or alveolitis)	116 912 (0.6)	113 (2.4)
Dementia	243 656 (1.3)	1025 (21.4)
Epilepsy	117 859 (0.6)	55 (1.2)
Heart failure	323 642 (1.7)	616 (12.9)
Learning disability or Down syndrome	129 057 (0.7)	34 (0.7)
Liver cirrhosis	40 134 (0.2)	30 (0.6)
Lung or oral cancer	36 752 (0.2)	50 (1.0)
Motor neurone disease, multiple sclerosis, myasthenia, or Huntington disease	11 735 (0.1)	13 (0.3)
Parkinson disease	67 801 (0.3)	139 (2.9)
Peripheral vascular disease	121 872 (0.6)	120 (2.5)
Pulmonary hypertension or fibrosis	38 487 (0.2)	110 (2.3)
Prior fracture (hip, wrist, spine, or humerus)	14 338 (0.1)	18 (0.4)
Rheumatoid arthritis or SLE	193 386 (1.0)	154 (3.2)
SCID	6094 (0.03)	10 (0.2)
Sickle cell or severe combined immunodeficiency syndrome	1624 (0.01)	<10^a^
Stroke or TIA	372 933 (1.9)	410 (8.6)
Thrombosis or pulmonary embolus	1819 (<.01)	<10^a^
Schizophrenia	107 154 (0.6)	33 (0.7)
Type 1 diabetes	232 298 (1.2)	87 (1.8)
Type 2 diabetes	1 538 173 (7.9)	798 (16.7)
BMI		
Morbidly obese	399 690 (2.1)	70 (1.5)
Overweight	3 680 341 (18.9)	880 (18.4)
Ideal	3 178 187 (16.3)	986 (20.6)
Underweight	138 729 (0.7)	145 (3.0)
Obese	2 541 104 (13.0)	491 (10.3)
Missing	9 535 519 (49.0)	2209 (46.2)
Care home resident	105 589 (0.5)	478 (10.0)
Region		
West Midlands	2 080 092 (10.7)	568 (11.9)
London	2 117 506 (10.9)	442 (9.2)
Yorkshire and Humber	2 011 985 (10.3)	551 (11.5)
North West	2 549 572 (13.1)	683 (14.3)
East Midlands	1 822 155 (9.4)	467 (9.8)
South East	3 361 853 (17.3)	737 (15.4)
South West	2 211 007 (11.4)	454 (9.5)
North East	999 584 (5.1)	331 (6.9)
East of England	2 319 816 (11.9)	548 (11.5)

Abbreviations: BMI, body mass index (calculated as weight in kilograms divided by height in meters squared); COPD, chronic obstructive pulmonary disease; IMD, index of multiple deprivation; SCID, severe combined immunodeficiency; SLE, systemic lupus erythematosus; TIA, transient ischemic attack.

^a^ Results capped at 10 cases to preserve patient anonymity.

Age was the most important characteristic associated with the risk of postbooster COVID-19 death (Figure, panel A), with an HR of 31.3 (95% CI, 26.1-37.6) for an 80-year-old individual compared with a 50-year-old. Women were at lower risk than men (HR, 0.52; 95% CI, 0.49-0.55). Living in a care home or in a socioeconomically deprived area were also associated with increased risk of COVID-19 death (Figure, panel B). There was no association between the risk of COVID-19 death and ethnicity, except for those of Indian background, who were at slightly elevated risk compared with White individuals.

**Figure.**
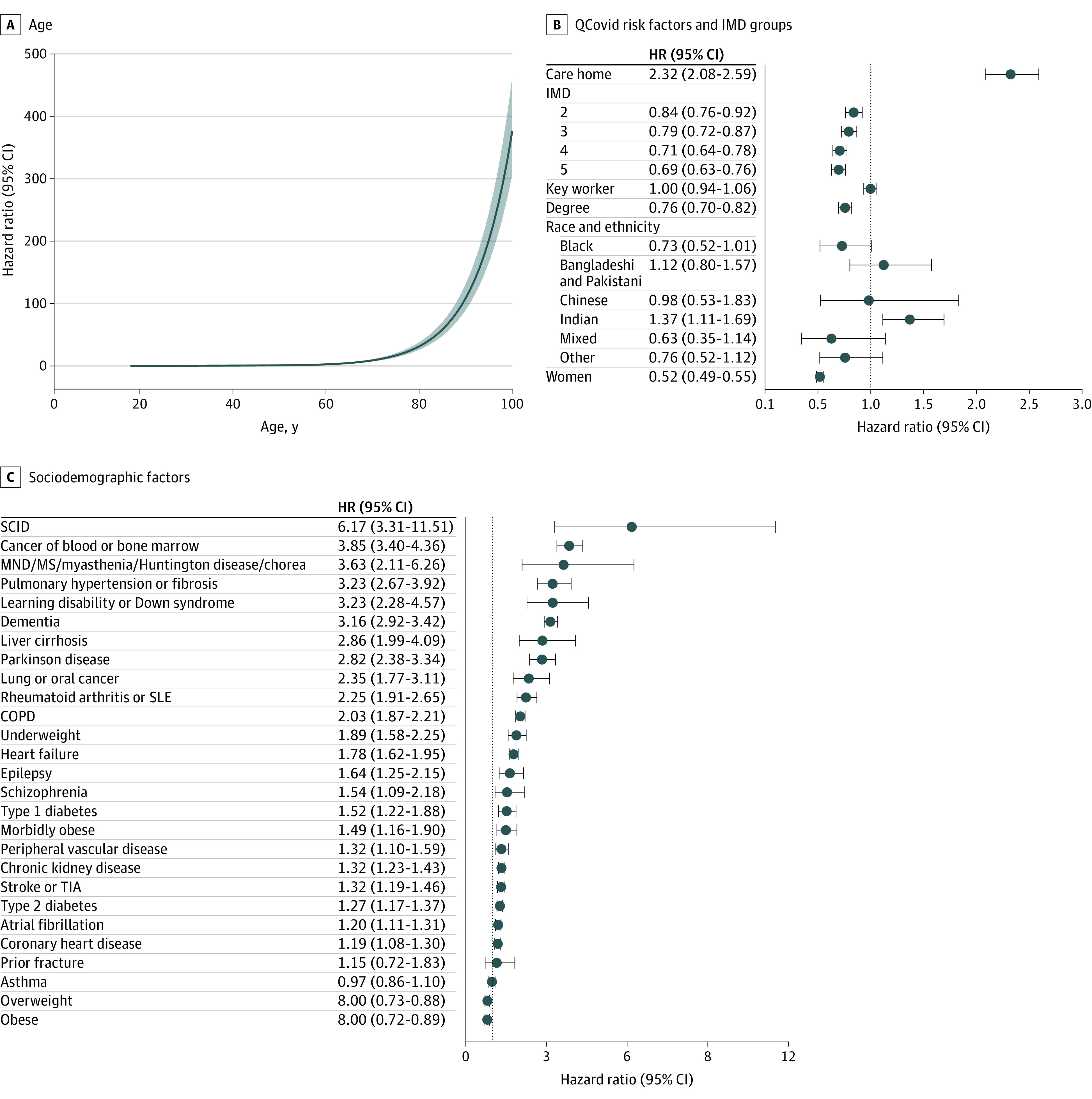
Risk Factors for Death From COVID-19 After Receiving a Booster Hazard ratios (HRs) were calculated with a Cox regression model. In panel A, the reference for HRs are men with healthy body mass index (BMI, which is calculated as weight in kilograms divided by height in meters squared) in index of multiple deprivation (IMD) quintile 1 for 50 days since last booster. In panels B and C, the dashed lines illustrate an HR of 1. Prior fracture includes fractures of the hip, wrist, spine, or humerus. COPD indicates chronic obstructive pulmonary disease; SCID, severe combined immunodeficiency; SLE, systemic lupus erythematosus; TIA, transient ischemic attack.

Most of the QCovid risk groups were associated with an increased HR of postbooster breakthrough death, except for congenital heart disease, asthma, and prior fracture (Figure, panel C). Risk was particularly elevated for people with severe combined immunodeficiency (HR, 6.2; 95% CI, 3.3-11.5). There were several conditions associated with HRs of greater than 3, including cancer of blood or bone marrow and dementia.

## Discussion

This study identified subpopulations that remain at increased risk of COVID-19 fatality after receiving their booster vaccine during the Omicron wave. Age remained the factor most associated with the risk of death, and men were at higher risk than women. The association with ethnicity was unclear and differed from previous studies, but this is likely to be due largely to the pronounced differences in vaccination uptake between ethnic groups in previous studies.^4^ Limitations of our study were that we only included data for the population living in England who were enumerated in the 2011 Census of England and Wales. The association between the QCovid risk groups and the risk of death were stronger in people who had received a booster and were infected by the Omicron variant compared with evidence from the Alpha and Delta period in doubly vaccinated individuals.^1,2,3^ The subpopulations with the highest risk should be considered a priority for COVID-19 therapeutics and further booster doses.^5,6^

## Notes

Supplement.
eMethods.
**eTable.** Sociodemographic Characteristics and Clinical Risk Factors

## References

[1] Hippisley-Cox J, Coupland CA, Mehta N, . Risk prediction of COVID-19 related death and hospital admission in adults after COVID-19 vaccination: national prospective cohort study. BMJ. 2021;374:n2244. doi:10.1136/bmj.n2244 34535466 PMC8446717

[2] Grange Z, Buelo A, Sullivan C, Moore E, Agrawal U, Boukhari K, McLaughlan I, Stockton D, McCowan C, Robertson C, Sheikh A, Murray JLK. Characteristics and risk of COVID-19–related death in fully vaccinated people in Scotland. Lancet. 2021;398(10313):1799-1800. doi:10.1016/S0140-6736(21)02316-3 34756204 PMC8553268

[3] Agrawal U, Katikireddi SV, McCowan C, . COVID-19 hospital admissions and deaths after BNT162b2 and ChAdOx1 nCoV-19 vaccinations in 2.57 million people in Scotland (EAVE II): a prospective cohort study. Lancet Respir Med. 2021.;9(12):1439-1449. doi:10.1016/S2213-2600(21)00380-5 34599903 PMC8480963

[4] Bosworth ML, Ahmed T, Larsen T, . Ethnic differences in COVID-19 mortality in the second and third waves of the pandemic in England during the vaccine roll-out: a retrospective, population-based cohort study. medRxiv. Preprint posted online February 15, 2022. doi:10.1101/2022.02.14.22270940 PMC9826727 36617562

[5] Emanuel EJ, Persad G, Upshur R, . Fair allocation of scarce medical resources in the time of COVID-19. N Engl J Med. 2020:382(21):2049-2055. doi:10.1056/NEJMsb2005114 32202722

[6] Reis G, Silva EASM, Silva DCM, Thabane L, . Effect of early treatment with ivermectin among patients with COVID-19. N Engl J Med. 2022;386(18):1721-1731. doi:10.1056/NEJMoa2115869 PMC9006771 35353979

