# Error in Table

**DOI:** 10.1001/jamanetworkopen.2022.38101

**Published:** 2022-09-29

In the Research Letter titled “Evaluation of Risk Factors for Postbooster Omicron COVID-19 Deaths in England,”^1^ published September 8, 2022, there was an error in a row label of the Table. The row with data for patients with sickle cell was labeled as results for sickle cell and severe combined immunodeficiency syndrome. Severe combined immunodeficiency syndrome has been removed, and this article has been corrected.^1^
