# Supplementary material for: Evaluation of Risk Factors for Postbooster Omicron COVID-19 Deaths in England
Source: JAMA Netw Open. 2022 Sep 8;5(9):e2233446. doi: 10.1001/jamanetworkopen.2022.33446 (PMC9459656; doi:10.1001/jamanetworkopen.2022.33446)
Supplement: Supplement. — eMethods. eTable. Sociodemographic Characteristics and Clinical Risk Factors [file jamanetwopen-e2233446-s001.pdf]

Supplemental Online Content

Nafilyan V, Ward IL, Robertson C, Sheikh A; for the National Core Studies—Immunology Breakthrough Consortium. Evaluation of risk factors for postbooster Omicron COVID-19 deaths in England. *JAMA Netw Open.* 2022;5(9):e2233446. doi:10.1001/jamanetworkopen.2022.33446

eMethods.

eTable. Sociodemographic Characteristics and Clinical Risk Factors

This supplemental material has been provided by the authors to give readers additional information about their work.

© 2022 Nafilyan VE et al. *JAMA Network Open.*

eMethods.

We used data from the Office for National Statistics Public Health Data Asset (PHDA), a population-level linked dataset combining the 2011 Census, mortality records, primary care records from the General Practice Extraction Service (GPES) data for pandemic planning and research, hospitalization from Hospital Episode Statistics (HES) and national vaccination data from the National Immunisation Management Service (NIMS). NIMS includes all vaccinations administered for all persons residing in England since the vaccination program started on December 8, 2020.

To obtain National Health Service (NHS) numbers, the 2011 Census was linked to the 2011-2013 NHS Participant Registers. Of the 53,483,502 Census records, 50,019,451 were linked deterministically. 555,291 additional matches were obtained using probabilistic matching (overall linkage rate: 94.6%). All subsequent linkages were conducted using NHS number. The ONS Public Health Data Asset include data on 35 million adults, which corresponds to an estimated 79% of the population of England in 2020 (calculated using official population estimates by age produced by the Office for National Statistics [1]).

Our study population included adults (18-100 years old) living in England who had received three Covid-19 vaccinations for at least 14 days on December 31, 2021.

The outcome was time to death involving Covid-19, defined as any death with codes U071 and U072 recorded anywhere on the death certificate, occurring between January 1 and March 16, 2022.

The predictors included in the model socio-demographic characteristics and clinical risk factors. The medical conditions were derived using primary care based on the definitions used by the QCovid2 risk prediction model. The QCovid risk prediction model was used by the NHS in the UK to identify clinically extremely vulnerable individuals. The model has been validated previously using the PHDA [1] and data from Scotland.[2] Models were also adjusted for time since booster dose. All predictors included in the model are summarized in Supplementary Table 1. Missing items for Census characteristics were imputed using nearest-neighbour donor imputation, the methodology employed by the Office for National Statistics across all 2011 Census variables. Missing BMI information was handles by including an 'Unknown' category.

A Cox regression model was used to estimate the hazard ratio (HR) of death involving Covid-19 for a range of socio-demographic characteristics and clinical factors, stratified by region. Time at risk started on January 1, 2022 and ended at time of death (either involving Covid-19 or not), or end of

---

1 https://www.ons.gov.uk/peoplepopulationandcommunity/populationandmigration/populationestimates/datasets/populationestimatesforukenglandandwalesscotlandandnorthernireland

© 2022 Nafilyan VE et al. *JAMA Network Open.*

study period (March 16, 2022). Non-Covid-19 deaths were censored. No information was available on whether people had moved out of the country.

eTable 1. Sociodemographic characteristics and clinical risk factors

**Variable**	**Coding**	**Source(s)**
Ethnicity	Bangladeshi or Pakistani, Black, Chinese, Indian, Mixed, White, Other	2011 Census
Age	Single year of age (restricted cubic spline)	2011 Census
Sex	Male, female	2011 Census
Residence type	Care home residence, other	2011 Census and 2019 NHS Patient register
Region	North East, North West, Yorkshire and the Humber, East Midlands, West Midlands, East, London, South East, South West	Postcodes from GDPPR and National Statistics Postcode Lookup (November 2019)
Index of Multiple Deprivation	Dummy variables representing quintiles of deprivation (1 most deprived; 5: least deprived)	Postcodes from GDPPR and English Indices of Deprivation, 2019
Highest qualification	Degree or above, other	2011 Census
Keyworker type	Key worker (education and childcare, national and local Government, public safety and national security, food and necessity goods, utilities and communications, transport, health and social care, key public services) or not	2011 Census
Time since third vaccination on Dec 31, 2021	Time in days (restricted cubic spline)	NIMS
Body mass index (kg/m^2)	< 18.5, 18.5 to < 25.0, 25.0 to < 30.0, 30.0 to < 40.0, $\geq$ 40.0, Unknown	GDPPR (8 December 2010 – 8 December 2020)
Health conditions	Binary flags for;<br>Asthma<br>Atrial Fibrillation<br>Cancer of Blood or Bone Marrow<br>Chronic Kidney Disease<br>COPD<br>Congenital Heart Problem<br>Coronary Heart Disease<br>Other rare chronic respiratory disorders (Cystic Fibrosis Bronchiectasis or Alveolitis)	GDPPR (2016 March 31 to 2021 March 31)

© 2022 Nafilyan VE et al. *JAMA Network Open.*

	Dementia<br>Epilepsy<br>Heart Failure<br>Learning Disability or Downs Syndrome<br>Liver Cirrhosis<br>Lung or Oral Cancer<br>Motor Neurone Disease or Multiple Sclerosis or Myasthenia or Huntington's Chorea<br>Parkinson's Disease<br>Peripheral Vascular Disease<br>Pulmonary Hypertension or Fibrosis<br>Prior Fracture<br>Rheumatoid Arthritis or SLE<br>SCID<br>Sickle Cell or Severe Combined Immunodeficiency Syndrome<br>Stroke or Tia<br>Thrombosis or Pulmonary Embolus<br>Schizophrenia<br>Type 1 diabetes<br>Type 2 diabetes	

© 2022 Nafilyan VE et al. *JAMA Network Open.*
